# Supplementary figures and images for: Proteasome inhibition triggers tissue-specific immune responses against different pathogens in C. elegans
Source: PLoS Biol. 2024 Mar 11;22(3):e3002543. doi: 10.1371/journal.pbio.3002543 (PMC10957088; doi:10.1371/journal.pbio.3002543)

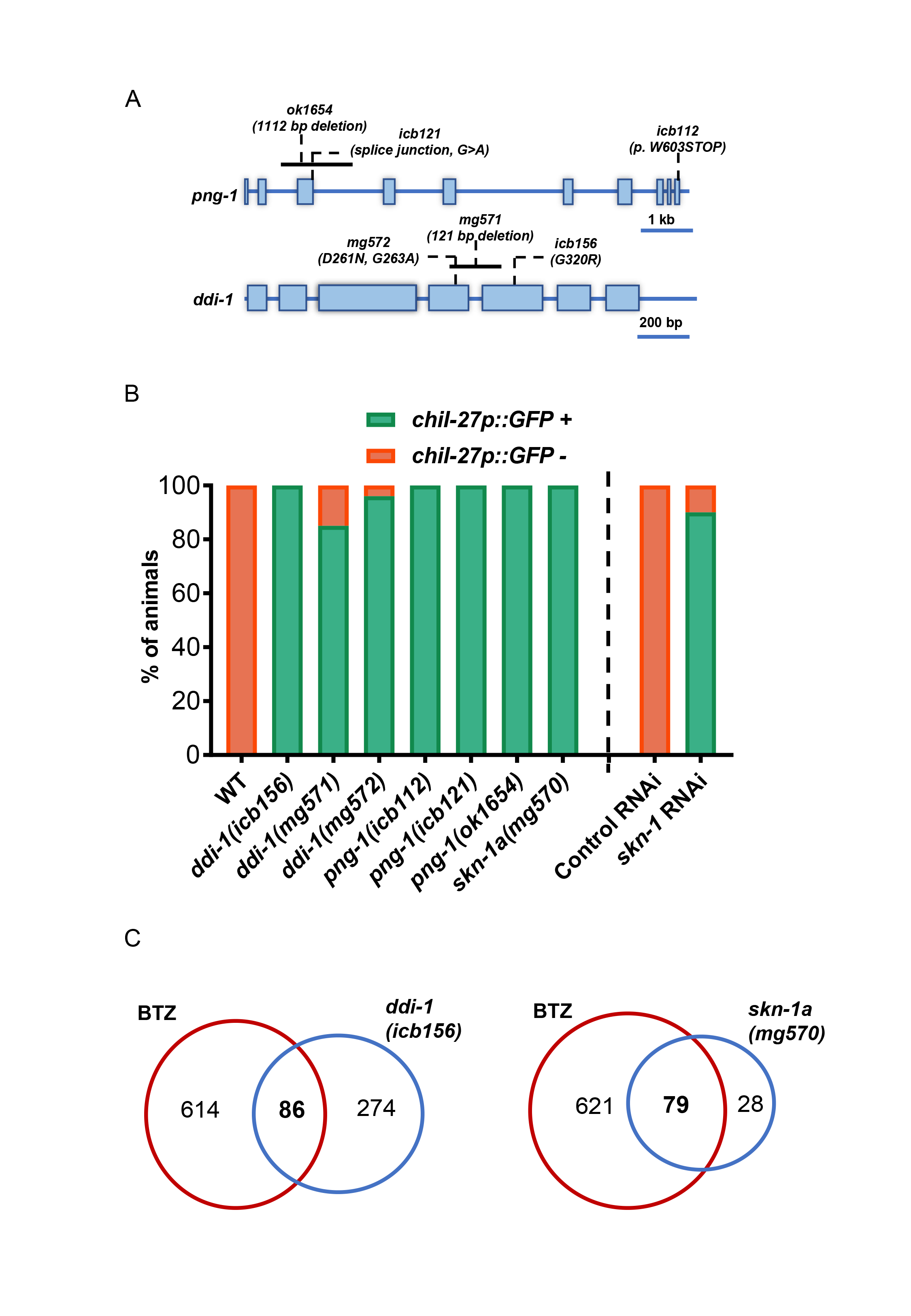

Supplement: S1 Fig — (A) Gene structure of ddi-1 and png-1 showing the positions of mutant alleles used in this study. (B) Quantification of induction of chil-27p::GFP in the mutants obtained from the EMS screen, ddi-1(icb156), png-1(icb112), png-1(icb121) along with active-site mutant of ddi-1(mg572), in-frame gene deletion of ddi-1(mg571), png-1(ok1654), skn-1a(mg570) and upon skn-1 RNAi (n > 50, ****p-value < 0.0001, *** p-value < 0.001 based on chi-square test). The numerical data for all 3 replicates is available in Supporting information S1 Data. (C) Venn comparisons showing significant overlap between up-regulated genes in the transcriptome of ddi-1(icb156) and skn-1a(mg570) mutant with bortezomib (BTZ)-treated animals (RF 6.0, p < 4.347e-43 and RF 18.6, p < 4.453e-88, respectively). (TIF) [file pbio.3002543.s001.tif]

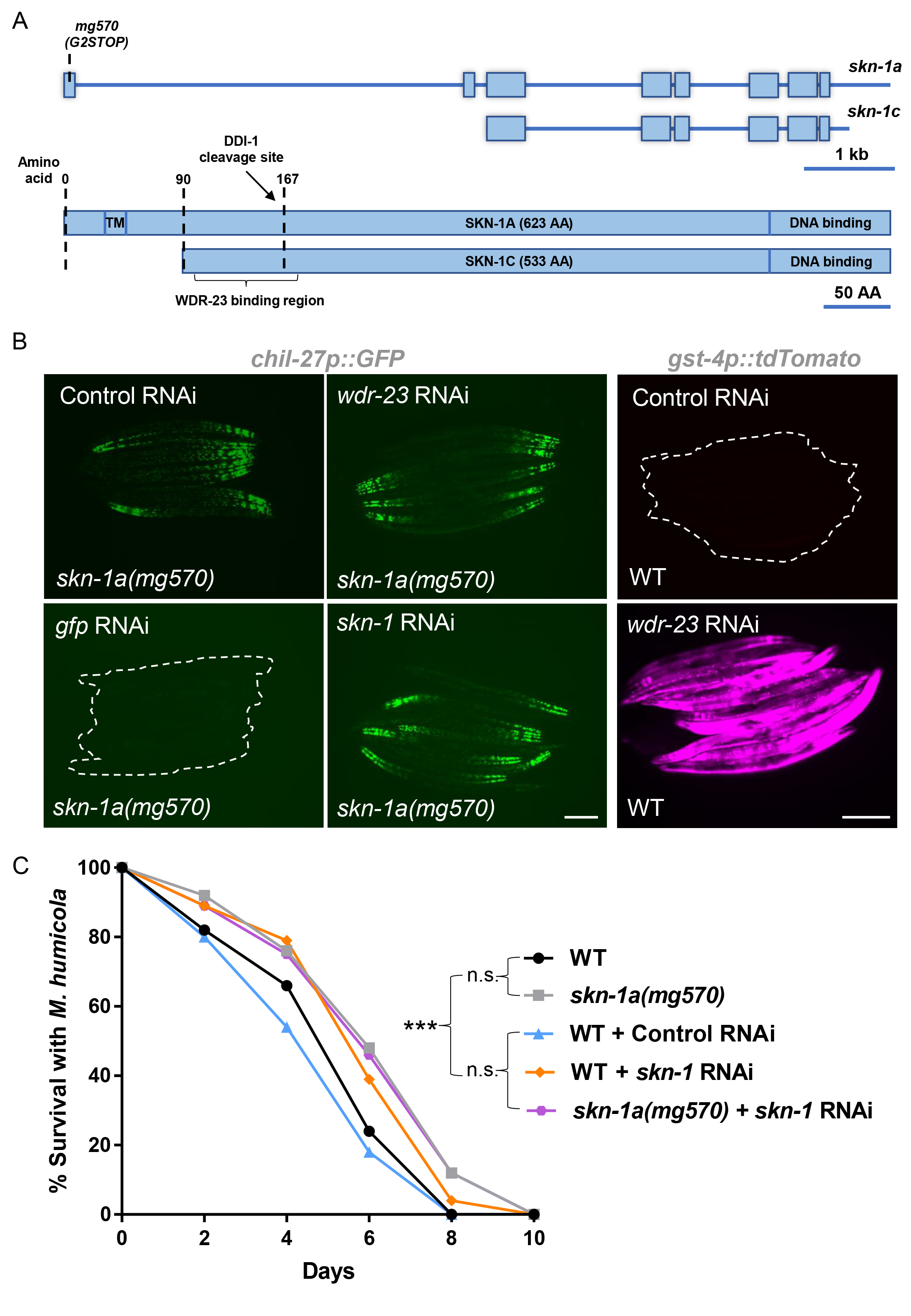

Supplement: S2 Fig — (A) Gene structure of skn-1a and skn-1c isoforms with protein domain organization. (B) Both loss (skn-1 RNAi) or gain (wdr-23 RNAi) of SKN-1C function does not affect chil-27p::GFP expression in skn-1a(mg570) (control RNAi) animals. skn-1a(mg570) animals are sensitive to RNAi as shown by the gfp RNAi treatment leading to complete loss of chil-27p::GFP expression (left panel). wdr-23 RNAi activates SKN-1C and leads to expression of gst-4p::tdTomato as shown in the right panel. (n > 50 per condition, performed in triplicates, representative image shown). Scale bar is 100 μm. (C) Survival curve of WT and skn-1a(mg570) animals treated and untreated with skn-1 RNAi in the presence of the oomycete M. humicola (n = 60 per condition, performed in triplicates, p < 0.001 based on log-rank test, a representative graph for one of the 3 replicates is shown). The numerical data for all 3 replicates is available in Supporting information S1 Data. (TIF) [file pbio.3002543.s002.tif]

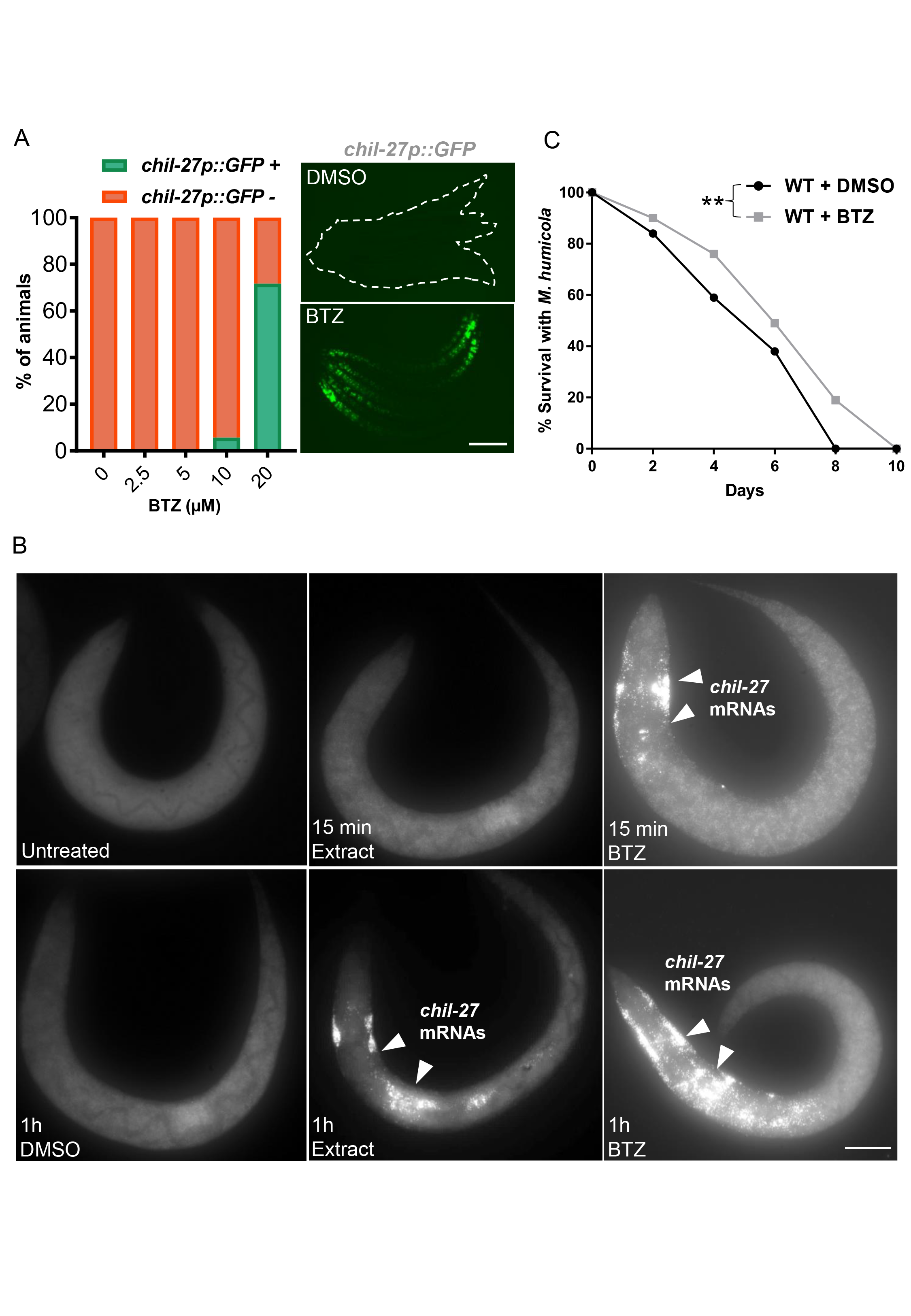

Supplement: S3 Fig — (A) chil-27p::GFP expression in WT animals treated with different doses of BTZ at early L4 stage for 24 h (n > 50 for each condition, performed in triplicates, representative image shown). (B) Z-stack of L2 stage WT animals showing expression of chil-27 mRNA upon 15 min and 1 h post extract and BTZ treatment. Scale bar is 100 μm in (A) and 10 μm in (B). (C) Survival curve of WT animals treated with DMSO or 20 μm BTZ in the presence of the oomycete M. humicola (n = 60 per condition, performed in triplicates, p < 0.001 based on log-rank test, a representative graph for one of the 3 replicates is shown). The numerical data for all 3 replicates of panels S3A and C is available in Supporting information S1 Data. (TIF) [file pbio.3002543.s003.tif]

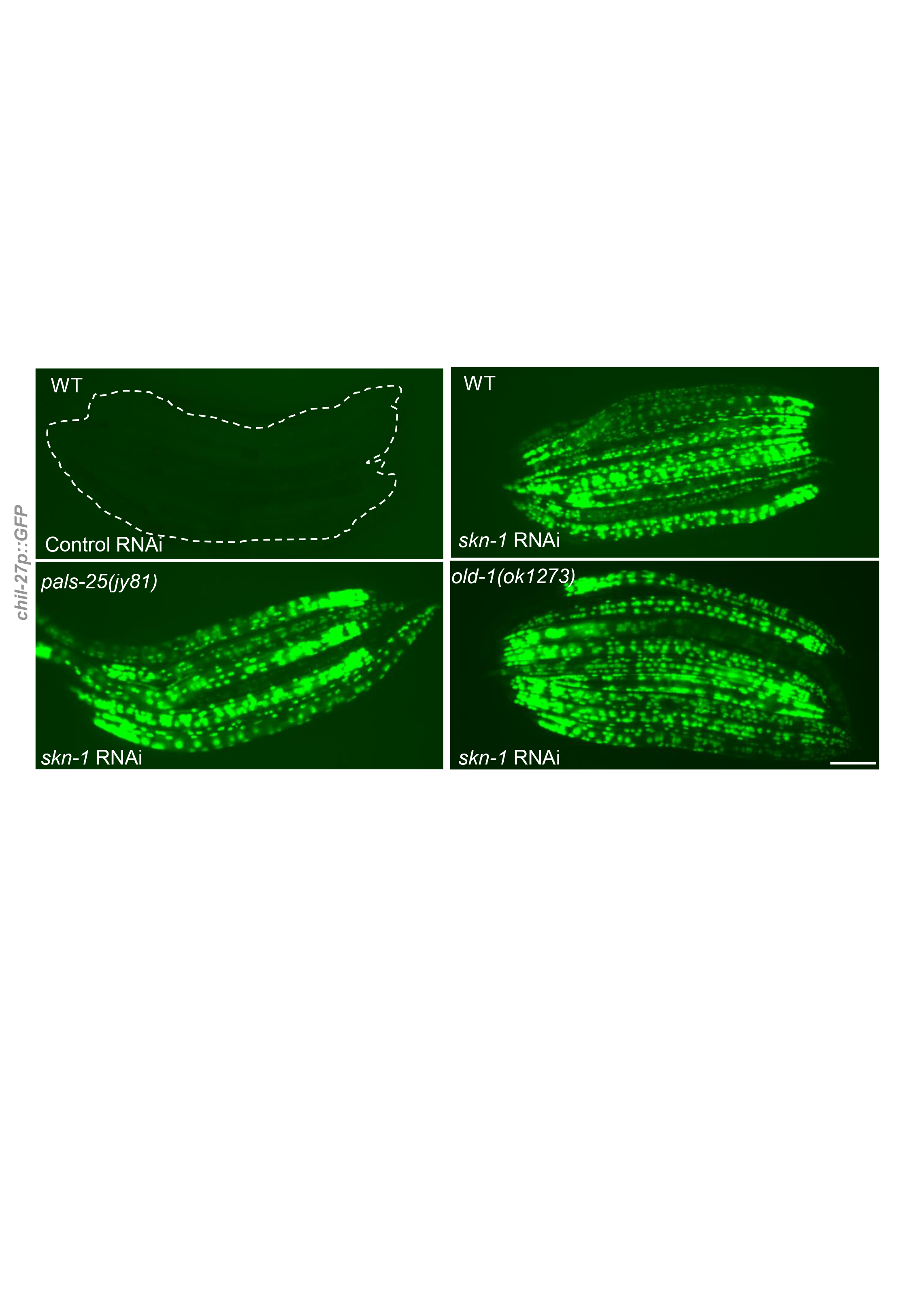

Supplement: S4 Fig — skn-1 RNAi induces chil-27p::GFP expression in pals-25(jy81) and old-1(ok1273) mutant animals. Scale bar for all panels is 100 μm, and n > 50 per condition, performed in triplicates, representative image shown. (TIF) [file pbio.3002543.s004.tif]

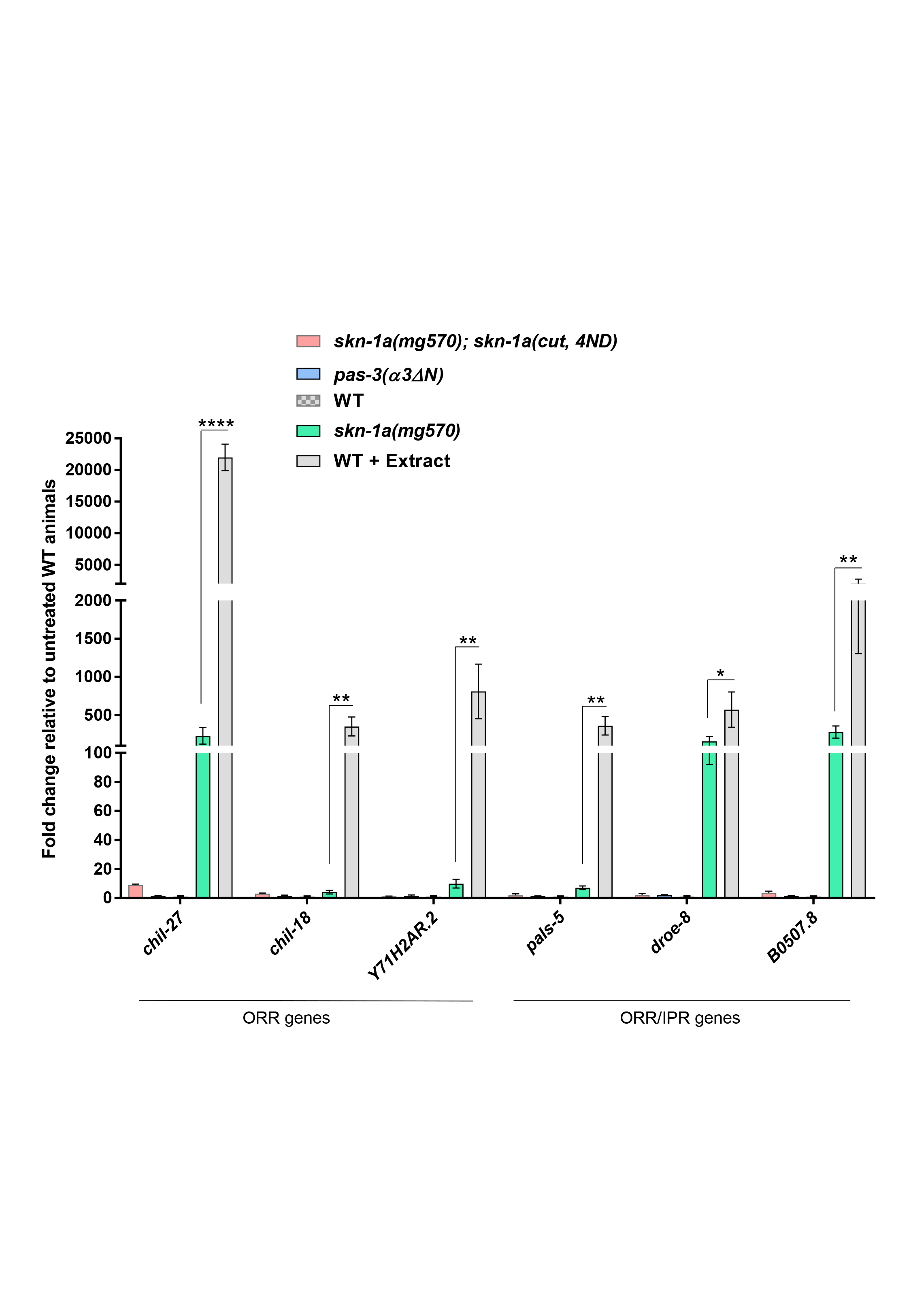

Supplement: S5 Fig — In the absence of extract treatment in animals with constitutive expression of the activated form of SKN-1A[skn-1a(cut, 4ND) in skn-1a(mg570)] or constitutive activation of the proteasome [pas-3(α3ΔN)] no expression of ORR genes was observed similar to wild-type animals. Note that ORR gene expression in skn-1a(mg570) mutants is lower compared to extract-treated wild-type animals (*p < 0.05, **p < 0.01, ****p < 0.0001 based on unpaired t test). The numerical data for all 3 replicates is available in Supporting information S1 Data. (TIF) [file pbio.3002543.s005.tif]

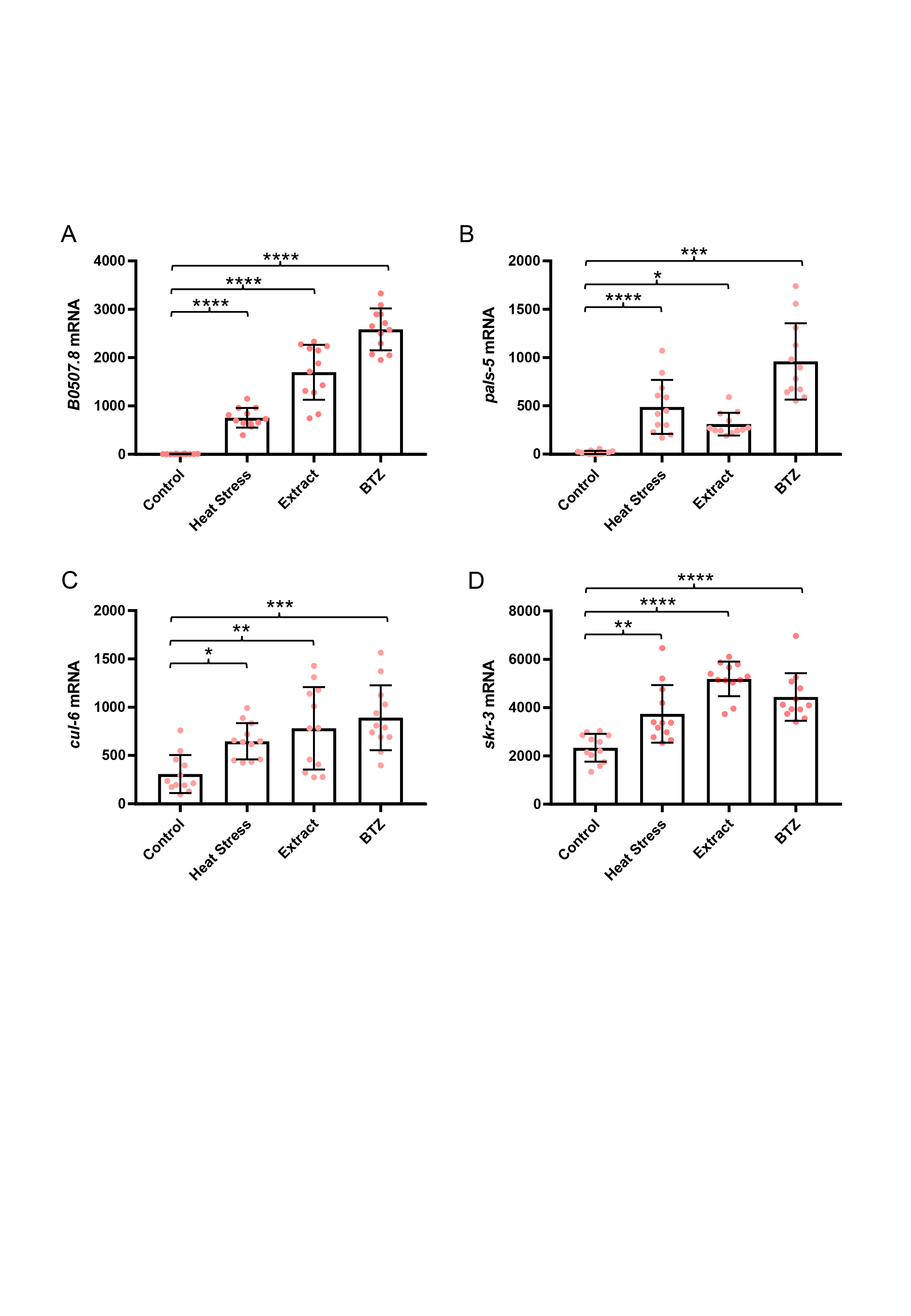

Supplement: S6 Fig — Whole animal mRNA quantification by smFISH for 4 genes, namely, B0507.8 (A), pals-5 (B), cul-6 (C), and skr-3 (D) in L2-stage animals subjected to prolonged heat stress (30°C for 24 h), extract treatment (4 h), and 20 μm BTZ treatment (2 h) (n = 12 in each condition and one-way ANOVA and Tukey’s multiple comparison test was used to assess statistical significance, *p < 0.05, **p < 0.01, ***p < 0.001, ****p < 0.0001). The numerical data for graphs A–D is available in Supporting information S1 Data. (TIF) [file pbio.3002543.s006.tif]

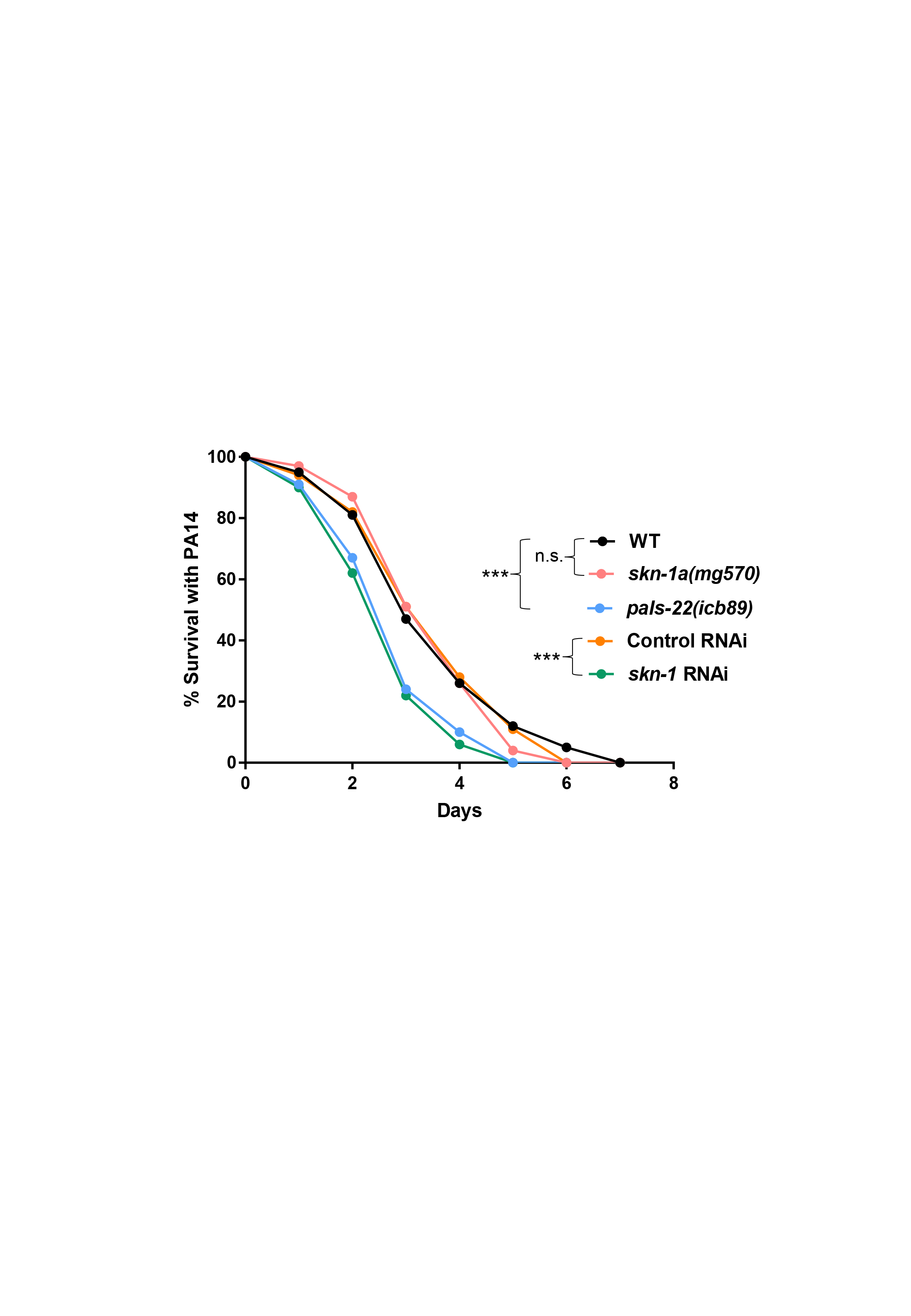

Supplement: S7 Fig — Survival analysis of adult C. elegans on PA14 where bacteria was spread on the plate (n = 90 per condition, performed in triplicates, p < 0.001 based on log-rank test, a representative graph for one of the 3 replicates is shown). The numerical data for all 3 replicates is available in Supporting information S1 Data. (TIF) [file pbio.3002543.s007.tif]
