# Supplementary material for: Proteasome inhibition triggers tissue-specific immune responses against different pathogens in C. elegans
Source: PLoS Biol. 2024 Mar 11;22(3):e3002543. doi: 10.1371/journal.pbio.3002543 (PMC10957088; doi:10.1371/journal.pbio.3002543)
Supplement: Attachment — Submitted filename: Response to reviewers.pdf [file pbio.3002543.s011.pdf]

We thank all the reviewers for providing thoughtful comments to help us improve our manuscript. Here is our point-by-point response to all comments.

Reviewer 1

This paper is full of superb surprises. First the screen for the genetic pathway that activates a chitinase response to an oomycete infection surprisingly finds two of the 3 components of the skn-1a proteasomal response pathway. It demonstrates how a great GFP screen can rather instantly tell the authors what pathway they are studying. The involvement of png-1 and ddi-1 and skn-1a was not AT ALL predictable from first principles of innate immunity. So the authors find themselves in a superb intersection between semi mature fields. And it is highly interesting that proteasome homeostasis is a central player in response to oomycete infection. The rescue of the skn-1a resistance in hypodermis in figure 3 is convincing.
Thank you for your comments

The smFish in Figure 5 was really difficult for me to discern. It is not a color blind issue. Perhaps better labeling would "sell" the commonality of the response to the various pathogens and BTZ.
We have improved the labelling and presentation of this figure and legend.

SKN-1A is one of the rare transcription factors that goes to the ER, an organelle at the center of secreted protein responses to pathogens. So the finding that skn-1a is at the center of ORR is really cool. Perhaps another sentence in the discussion to get the readers up to speed.
We have included in the discussion a reminder to the readers about the unique feature of SKN-1A being an ER localized transcription factor in the discussion section (line 397).

Figure 1C is really supplemental....showing locations of alleles is only worthy of a main display figure if there is something interesting about locations or types of mutations.
We have moved Fig 1C to the supplement (Fig S1A).

Figure 1E Y axis should be labelled with "% survival w oomycete infection" (always aim to make Figures interpretable without having to read Figure legends for key hints).
We have modified the labelling of the figure as suggested.

Figure2C is not visible to the Red Green Color blind without activating color blind accessibility triggers.
We have fixed this issue as well as all other cases throughout the manuscript.

Reviewer 2

This interesting manuscript describes a homeostatic relationship between two responses to eukaryotic pathogens and the proteasome, with a key role being played by SKN-1A/Nrf1, the master transcriptional regulator of proteasome subunit gene transcription. The authors uncovered this relationship through a genetic screen that led them to regulators of SKN-1A. The results are of interest not only to the

pathogenesis and immunity fields but also generally, because they uncover a pathway of crosstalk between immune defenses and the ubiquitin proteasome system. An additional benefit is that the paper defines a new example of evidence that discriminates between the functions of SKN-1A/Nrf1 and SKN-1C/Nrf2, which are expressed from alternatively spliced isoforms of the same gene. Studies in this area frequently conflate or confuse the functions of these two proteins, which are each important in aging, stress resistance, metabolism, and now immunity. The manuscript is well-written and the data are of high quality, with an important exception noted below.
Thank you for your comments.

The time has past (or should be past) when it is sufficient to present pictures of grouped worms or individual images to assess gene expression. The former is highly subjective and unreliable, and the latter (i.e. Fig. 5) gives us no indication of the numbers analyzed or reproducibility. This is no longer allowed at most top journals. I hesitate to ask the authors to repeat all of these experiments (the ideal situation), but they really should take significant steps in this direction like repeating ANY experiments that are not clear on/off situations with scoring and quantification, statistics. They should also indicate the numbers of animals analyzed and consistency of results for ALL such experiments. In today's world some experiments should be scored blindly, as well.
We certainly agree with the reviewer. All information about quantification and statistics is now included in the figures and their corresponding legends or supplement. In particular, quantification of Fig. 5 is shown in Fig. S6.

It is interesting that the authors have implicated proteasomal homeostasis in these immunity pathways, but the paper would have more impact if they determined what regulator(s) was/were targeted by the proteasome. The Troemel lab has implicated the transcription factor ZIP-1 in the IPR but this, oddly, was not mentioned. Could this factor or another candidate be targeted by the proteasome? Sorting this out is not necessarily essential for this paper but would greatly strengthen it.

The reviewer brings up a good point and we are actively searching for the nature of these factors. In terms of ZIP-1, we mention it as a candidate proteasomally regulated factor for intestinal activation of IPR in the discussion (line 475). However, we know that a separate factor is playing a role in the epidermis, as ZIP-1 is not required for activation of the ORR in the epidermis.

In addition to the experiments performing wdr-23 RNAi, a complementary approach for ruling out functions for SKN-1C would be to perform skn-1 RNAi in WT and skn-1a mutant strains in parallel. This should be applied to the gene expression and pathogenesis studies, or at least some of them. It is a direct way to address SKN-1C functions that could strengthen the claims made.
Thank you for this suggestion. We've now performed *skn-1* RNAi in WT and *skn-1a(mg570)* mutants and analysed both *chil-27p::GFP* expression as well as survival upon oomycete infection. There was no difference observed in constitutive *chil-27p::GFP* expression of *skn-1a(mg570)* mutants upon *skn-1* RNAi (*see new figure S2B,C*). Similarly, oomycete-resistance phenotype of *skn-1a(mg570)* mutants was unaffected by *skn-1* RNAi *(see new figure S2C).* Also, we confirmed the RNAi

sensitivity of *skn-1a(mg570)* mutants, and the efficacy of *wdr-23* RNAi treatment and these results are included in Figure S2B.

A recent study (PMID: 36598980) implicated SKN-1A in fat/lipid metabolism and showed that its absence increases stored fat levels. Given that availability of specific fats has been implicated in other immune responses, this should be considered or at least discussed. Were any SKN-1A-regulated fat metabolism genes picked up in the datasets described here?
Thank you for this suggestion. We compared the list of SKN-1A-regulated fat metabolism genes described in Castillo-Quan *et al.,* Science Advances 2023) with the datasets in our study and found only *fat-5* expression to be upregulated upon oomycete extract treatment (ORR), BTZ treatment and loss-of-function of *ddi-1(icb156).* Given than oleic acid production has been linked with both activation of SKN-1A and survival of *fat-6(-);fat-7(-)* double mutants on PA14 (Anderson *et al*., PLOS Pathogens 2019), we have included a comment (lines 347-349) that this might an interesting area to pursue in the future.

As an alternative model, is it known whether the genes involved in these immune responses have conserved recognition sequences that would be bound by SKN-1A and repressed?

To test this model, we compared the list of ORR and IPR genes with SKN-1 target genes identified by ChIP (Staab *et al*., 2013). We did not find any overlap between the two datasets suggesting that there's minimal likelihood of immune genes to be suppressed by the binding of SKN-1A. It will be interesting to revisit this idea in the future once direct targets are known for all different SKN-1 isoforms.

Very minor: The Lehrbach 2016 eLife paper is a landmark in this area and should be cited earlier, in the introduction.
Agreed. We have now included a citation for the mentioned paper in the introduction (line 99).

Reviewer 3

The conserved SKN-1A/Nrf1 transcription factor regulates proteasome subunit gene expression to ensure adequate proteasome function. In this study, Grover et al show that mutations that disrupt SKN-1A/Nrf1 in C. elegans lead to misregulation an innate immune signaling pathway called the oomycete recognition response (ORR). Given previous work suggesting that both the ORR, and a related immune response to intracellular pathogens (the IPR), are activated in animals experiencing proteasome dysfunction, this finding prompted the authors to explore the relationship between skn-1, the proteasome, and immunity in C. elegans.

Overall, this work advances the field by making a novel link between SKN-1/Nrf and innate immune regulation and refines our understanding of the interplay between the proteasome and C. elegans immune regulation that had been hinted by previous studies. The interest to a wider scientific audience is somewhat limited without mechanistic insight into the regulatory mechanism(s) that link proteasome dysfunction to innate immune control, but addressing this issue would be better left to future studies. I recommend that this work is suitable for publication in PLoS

Biology with minor changes and limited additional experiments to address the comments listed below.
Thank you for your comments

The authors show that skn-1a mutants show increased expression of ORR genes and resistance to oomycete infection and propose that both of these effects are the result of impaired proteasome function in skn-1a mutants. However, SKN-1A/Nrf1 may also regulate other genes aside from the proteasome subunits. The argument that the skn-1a mutants' resistance to oomycete infection results from reduced proteasome function (rather that misregulation of other skn-1a target genes) would be strengthened by testing the effect of BTZ treatment or proteasome subunit RNAi on resistance to oomycete infection.
We have now performed oomycete infection assays with BTZ-treated animals and found that they can show enhanced resistance *(see new figure S3C)*. To perform these assays, we pre-treated L4 animals with BTZ for 24 hours before exposing them to the pathogen to minimise potentially confounding effects of chemical inhibitors on eukaryotic pathogens. Within the time that was given to us to revise the manuscript, we chose not to prioritise RNAi treatment of proteasome subunits because these have recently been ascribed additional roles beyond protein degradation (Olaitan *et al*. PLOS Genetics 2018; Fernando *et al*. Front. Cell Dev. Biol. 2022).

Related to the above point - knock-down of proteasome subunits by RNAi sensitizes C. elegans to infection by N. parisii (Bakowski et al 2014, PMID: 24945527), suggesting that the proteasome is required for defense against infection by this pathogen. In this study, the authors find that skn-1a mutants (in which proteasome subunit expression is reduced) are resistant to N. parisii infection. It would be interesting for the authors to include a discussion of this discrepancy. One interesting possibility could be that the proteasome has protective roles in combatting infection, in addition to a role in regulating immune responses. In that case, differing degrees of proteasome dysfunction may have different results on immunity. I.e., mild proteasome dysfunction might enhance pathogen resistance through ORR/IPR activation, whereas severe proteasome dysfunction could increase sensitivity by disrupting proteasome-dependent immune defenses. This could be tested by comparing the effects of different BTZ concentrations on C. elegans' sensitivity to infection.
We agree with the reviewer that the extent of proteasome dysfunction is likely to determine the activation of ORR/IPR. We have some indirect evidence to support this. For example, *skn-1a(mg570)* mutants do not induce *chil-27p::GFP* at the early larval stages (while high dose of BTZ treatment can trigger *chil-27p::GFP* induction). Furthermore, *skn-1a(mg570)* mutants do not exhibit resistance to microsporidia at the L1 stage. These observations suggest that sufficient proteasome dysfunction may need to accumulate before immune responses are activated. We have added a comment in the discussion (lines 413-419) about the possibility that the extent of proteasome dysfunction may determine the activation of ORR/IPR.

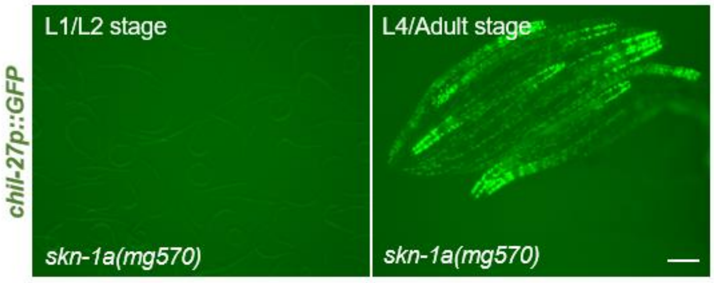

L1/L2 stage *skn-1a(mg570)* animals do not show robust constitutive expression of *chil-27p::GFP* unlike L4/adult stage animals.

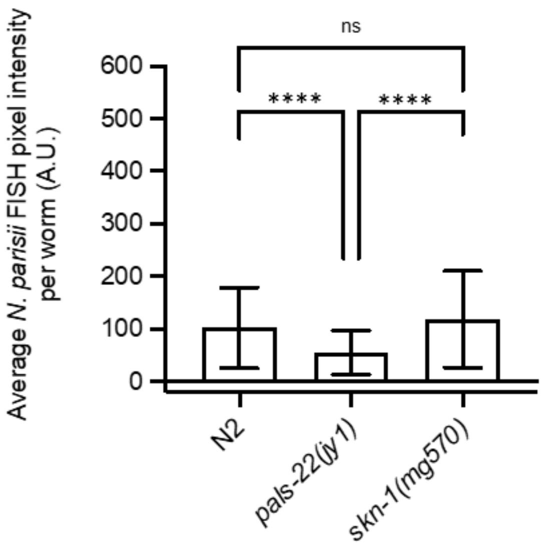

Pathogen load assay for L1 stage animals infected with *N. parisii*. The graph shows combined data from three independent repeats (n=150). **** $p < 0.0001$, Kruskal Wallis with Dunn's multiple comparisons.

Thank you for the perceptive comment on how we can reconcile why proteasome subunit RNAi may have led to susceptibility to *N. parisii* in a previous study in light of the new results obtained using *skn-1a* mutants.

As loss of *pas-5* and *rpn-2* is associated with growth and development defects (Simmer *et al*., PLOS Biology 2003; Green *et al*., Cell 2011), in the previous study by Bakowski *et al* 2014 diluted RNAi was used only for 24 hours before infection with *N. parisii*. It is therefore possible that diluted RNAi may have only led to mild proteasome dysfunction that was insufficient in this case to activate the immune response.

There are alternative explanations of course through the feedbacks in the pathway or other indirect effects within the complex context of infection. For example, RNAi of either of the core proteasomal subunit genes including *pas-5* has been shown to activate SKN-1 (Kahn *et al*., Biochem J. 2008), thus mild RNAi treatment might cause at least transiently activation of proteasome and reduced activation of IPR upon *N. parisii* exposure and consequently mild increase in pathogen load. Bakowski *et al.* also showed that as a part of microsporidia pathogenesis in *C. elegans*, pathogen cells are targeted by host ubiquitin early during infection. This suggests that the increased load of *N. parisii* observed previously may be attributed to changes in the ability of cells to ubiquitinate the pathogen independently of the IPR. For example, both PAS-5 and

RPN-2 have been shown to regulate expression of *rpn-11* (Li *et al*., PLOS Genetics 2011), which is the main deubiquitinating enzyme of the proteasome (Verma *et al*., Science 2002).

The evidence that hyperactivation of the proteasome attenuates immune responses (fig 3D) needs to be strengthened and/or interpreted more cautiously: Firstly, in this experiment the expression of ORR genes in skn-1a(cut, 4nd) and pas-3ΔN animals treated with extract is compared to WT animals treated with extract as a control. The authors conclude that: 'hyperactivity of the proteasome significantly inhibits oomycete extract-mediated chil-27 gene induction.' However, the data presented do not distinguish between the possibility that animals with hyperactive proteasomes are defective in induction of chil-27, Vs simply show reduced chil-27 expression levels regardless of exposure to extract. The levels of chil-27 mRNA must be compared between WT and both skn-1a(cut, 4nd) and pas-3ΔN in the absence of oomycete extract. If chil-27 mRNA level is identical in the absence of extract, this supports the statement that induction is defective. But if uninduced chil-27 mRNA level is also reduced, this would suggest that proteasome hyperactivation generally reduces chil-27 mRNA levels (but does not necessarily disrupt the pathogen-responsive signaling pathway that induces chil-27 and other ORR genes in response to extract).
We did not include the data in the previous version because in the absence of extract *chil* genes (as well nearly 60% of other ORR genes) have low or no detectable expression (Fasseas *et al*., Cell Reports 2021). We have now included the qPCR data without extract treatment in the supplement *(see new figure S5)*. Please note that the strain denoted by *skn-1a[cut, 4ND]* is *skn-1a(mg570)* loss-of-function mutant overexpressing the activated form of *skn-1a* under a ubiquitous promoter. As a result, this strain does exhibit some residual activation of ORR genes although this is greatly reduced in comparison to *skn-1a(mg570)* mutants or extract-treated WT animals.

Secondly, animals with hyperactive proteasomes (skn-1a(cut, 4nd) and pas-3ΔN) express ORR genes at relatively high levels compared to uninduced WT in response to oomycete extract exposure, even if expression levels are slightly lower than in similarly induced WT animals. So it is unclear whether this change in immune gene expression has any functional significance. This should be addressed by measuring the effect of skn-1a(cut, 4nd) and pas-3ΔN on survival following oomycete infection.
We agree with the reviewer that strains with hyperactive proteasomes still exhibit high induction of ORR genes upon oomycete extract exposure that is only slightly reduced in comparison to WT animals. This induction is beyond what is seen in *skn-1a(mg570)* mutants showing the oomycete resistance phenotype, so it is unlikely that strains with hyperactive proteasomes would exhibit any increase in susceptibility towards the pathogen. We have not been able to directly test this hypothesis because strains with hyperactive proteasomes show significant fitness defects making infection assays in these backgrounds inconclusive.

In Fig 5, prolonged heat-stress was used as a proxy for inducing the IPR, could the authors please explain why this was used instead of pathogen exposure? Since prolonged heat stress would presumably affect all tissues of the animal, why does prolonged heat stress only activate the intestinal IPR but not the hypodermal ORR?
We have added a sentence (lines 352-353) to clarify that heat stress is used as a proxy for activating the IPR in the intestine (Reddy et al. Current Biology 2017), similar to how oomycete extract treatment is used to activate the ORR in the epidermis

(Fasseas et al. Cell Reports 2021). We used heat stress and oomycete extract for reasons of consistency because every worm responds to prolonged heat stress/oomycete extract exposure, whereas not all worms upregulate IPR/ORR gene expression due to variable infection. While it is intuitive that heat stress may affect all tissues of an animal, tissue-specificity in the heat shock response has been previously reported in *C. elegans* (Guisbert *et al*., PLOS Genetics 2013). Furthermore, the intestine appears to be a key tissue for thermotolerance (Reddy *et al*., Current Biology 2017; Panek *et al*., PNAS 2020), for example expression of *pals-22* in the intestine, but not the epidermis, has been reported to rescue the thermotolerance phenotype observed in *pals-22* mutants.

The differing phenotype caused by skn-1(RNAi) vs the skn-1a mutant is used to infer that the role of skn-1 in PA14 resistance is more likely to be associated with SKN-1C isoform. But these data are equally consistent with the possibility that SKN-1A and SKN-1C isoforms are redundant for this function. The text should be modified to include this possibility.
We agree with the reviewer and have modified the text as we cannot rule out that SKN-1A and SKN-1C may play a redundant role towards protection from PA14 (lines 385-386 and 456).

The idea that different skn-1 isoforms are 'pathogen-specific' should be supported by experiments to address the role of SKN-1C in resistance to eukaryotic pathogens. This could be done by testing whether skn-1(RNAi) - ie kockdown of both skn-1a and skn-1c - alters the resistance of WT and/or the hyper-resistant skn-1a mutants to oomycete or N. parisii infection.
To address this point, we've performed oomycete infection assays on *skn-1* RNAi treated WT animals and *skn-1a(mg570)* mutants. We found that *skn-1a(mg570)* mutants treated with *skn-1* RNAi still show enhanced resistance towards *M. humicola (see new figure S2C)*

Typo: On line 103, the NGLY1 gene is referred to as NGLY
Fixed

NRF1 should also be referred to as NFE2L1 (at least when NRF1 is first mentioned in the introduction at line 99) to avoid confusion with Nuclear Respiratory Factor 1, which can also be referred to by the acronym NRF1.
Fixed
