# Supplementary material for: Proteasome inhibition triggers tissue-specific immune responses against different pathogens in C. elegans
Source: PLoS Biol. 2024 Mar 11;22(3):e3002543. doi: 10.1371/journal.pbio.3002543 (PMC10957088; doi:10.1371/journal.pbio.3002543)
Supplement: Attachment — Submitted filename: Response to reviewers.docx [file pbio.3002543.s012.docx]

Thank you for your patience while we considered your revised manuscript entitled "Impairment of the SKN-1A/NRF1 proteasome surveillance pathway triggers tissue-specific protective immune responses against distinct natural pathogens in C. elegans" for publication as a Research Article at PLOS Biology. This revised version of your manuscript has been evaluated by the PLOS Biology editors and by the Academic Editor.
Based on our Academic Editor's assessment of your revision, we are likely to accept this manuscript for publication, provided you satisfactorily address the data and other policy-related requests stated below.

Thank you. We are happy to know that the editors found the revised manuscript suitable for publication.

In addition, we would like you to consider a suggestion to improve the title:

"Proteasome inhibition triggers tissue-specific immune responses against different pathogens in C. elegans"

We have changed the title of the manuscript as suggested.

As you address these items, please take this last chance to review your reference list to ensure that it is complete and correct. If you have cited papers that have been retracted, please include the rationale for doing so in the manuscript text or remove these references and replace them with relevant current references. Any changes to the reference list should be mentioned in the cover letter that accompanies your revised manuscript.

We have replaced reference 33 with the following:

Lo JY *et al*., WDR23 regulates NRF2 independently of KEAP1. PLoS Genet. 28;13(4):e1006762.

DATA POLICY: IMPORTANT - PLEASE READ

You may be aware of the PLOS Data Policy, which requires that all data be made available without restriction: http://journals.plos.org/plosbiology/s/data-availability. For more information, please also see this editorial: http://dx.doi.org/10.1371/journal.pbio.1001797

Note that we do not require all raw data. Rather, we ask that all individual quantitative observations that underlie the data summarized in the figures and results of your paper be made available in one of the following forms:

1) Supplementary files (e.g., excel). Please ensure that all data files are uploaded as 'Supporting Information' and are invariably referred to (in the manuscript, figure legends, and the Description field when uploading your files) using the following format verbatim: S1 Data, S2 Data, etc. Multiple panels of a single or even several figures can be included as multiple sheets in one excel file that is saved using exactly the following convention: S1_Data.xlsx (using an underscore).

2) Deposition in a publicly available repository. Please also provide the accession code or a reviewer link so that we may view your data before publication.

Regardless of the method selected, please ensure that you provide the individual numerical values that underlie the summary data displayed in the following figure panels as they are essential for readers to assess your analysis and to reproduce it:

Fig. 1D; Fig. 2C; Fig. 3A, D; Fig. 4C, D; Fig. S1B; Fig. S2C; Fig. S3A, C; Fig. S5; Fig. S6A-D and Fig. S7


NOTE: the numerical data provided should include all replicates AND the way in which the plotted mean and errors were derived (it should not present only the mean/average values).

Please also ensure that figure legends in your manuscript include information on WHERE THE UNDERLYING DATA CAN BE FOUND, and ensure your supplemental data file/s has a legend.

Numerical data for all the graphs mentioned above are included in an excel file as supporting information S1 Data and this has been mentioned in all respective figure legends.

Please ensure that your Data Statement in the submission system accurately describes where your data can be found.
